# Construction of Garlic Core Collections Based on the Agronomic Traits of 366 Germplasm Accessions

**DOI:** 10.3390/plants14193009

**Published:** 2025-09-28

**Authors:** Yunpeng Zhong, Chengyu Li, Qi Qiao, Xiaoxi Lu, Deyi Xie, Jun Mao, Fengyin Lu, Zhongjie Tang, Zhiyong Wu

**Affiliations:** Economic Crop Research Institute, Henan Academy of Agricultural Sciences, Zhengzhou 450002, China

**Keywords:** garlic, germplasm accessions, agronomic traits, core collection

## Abstract

In this study, 21 agronomic traits (14 quantitative and 7 qualitative) of 366 garlic germplasm accessions were analyzed using correlation, principal component, and cluster analyses. Genetic diversity was analyzed by the unweighted pair group method with arithmetic mean (UPGMA). Random (R), priority (P), and deviation (D) sampling methods were adopted to construct core collections, and their representativeness was evaluated. The resources exhibited rich genetic diversity and were divided into five categories. Three core collections, R1, P1, and D1, were constructed, and R1 and P1 conformed to core construction principles. A total of 13 R1 traits had higher variances and coefficients of variation, 15 P1 traits showed significant differences in variance and 20 had higher coefficients of variation, and 16 D1 traits exhibited significant differences in mean values compared to those in the original population. Therefore, the three core collections had good heterogeneity, and the P1 group achieved the greatest genetic variation. Based on the three sets, 90 garlic core collections were constructed, accounting for 24.59% of total resources. These core collections provide a scientific basis for the establishment and conservation of a garlic germplasm resource nursery and the breeding of new varieties.

## 1. Introduction

Garlic (*Allium sativum* L.) is an herbaceous plant belonging to the *Allium* genus of the *Liliaceae* family. It is not only an important condiment vegetable [1] but also a valuable medicinal plant [2,3]. Previous studies have shown that garlic originated in Central Asia. Its ancestor was domesticated, and then introduced to the Mediterranean Basin, India, and China before spreading to other parts of the world [4]. During the Han Dynasty of China, garlic was introduced to the Guanzhong region of China and subsequently cultivated on a large scale. Today, garlic cultivation has expanded to all continents except Antarctica [5]. As a typical vegetable with both medicinal and nutritional value, the bulb is the main edible organ of garlic. In addition to rich vitamin, carbohydrate, and mineral elements, garlic contains allicin, an important functional component, and can also accumulate high levels of selenium and germanium. These properties give garlic important medical and health effects, such as anti-bacterial, anti-inflammatory, blood sugar- and lipid-lowering, anti-cancer, heavy metal-protective, and age-delaying properties [6,7,8].

The global ex situ holdings of garlic accessions are abundant, with a total of over 5000 accessions according to incomplete statistics. China, India, the Czech Republic, Poland, the United States, Germany, and Russia are major countries with rich garlic germplasm resources, and each country conserves more than 400 accessions [9,10]. In China, ex situ conservation and ultra-low temperature conservation are the main methods for preserving garlic germplasm resources [9].

Garlic mainly reproduces asexually through bulbs, which greatly limits the breeding of new varieties. Although previous studies have reported fertile garlic resources in the northern region of the Tianshan Mountains and Central Asia that produce seeds through selfing or hybridization and that purple anthers are the main morphological characteristic of most male-fertile garlic resources [11,12], no new garlic varieties bred through sexual hybridization have been reported. During the flowering period of garlic, the aerial bulbs expand rapidly, causing extrusion and nutrient competition. Pollen mother cells gradually dry out during pollen grain formation, leading to complete drying of anthers before flowering. Even under artificial optimal conditions, garlic anthers fail to germinate pollen tubes. Additionally, high-frequency chromosomal structural variations in pollen mother cells collectively result in sterile pollen grains, preventing the breeding of new varieties through sexual hybridization [13,14].

The concept of a core collection was first proposed by Frankel in 1984 [15]. It refers to a subset of accessions selected from a collected resource population, with a limited quantity (usually 5–20% of the total accessions) that represents the genetic diversity of the entire collection. The construction of one or more core collections compressed the number of original germplasm accessions by removing redundant materials with similar genetic backgrounds without reducing genetic diversity, reduced the cost of germplasm conservation and reproduction renewal, and improved the efficiency of germplasm conservation [16,17]. Moreover, the construction of core collection should be carried out before in-depth evaluation of germplasm accessions [18]. Since the core collection contains the main genetic variation of the germplasm accession, it provides breeders with more detailed information, and can be used to quickly screen parent materials related to target traits, reduce the blindness of breeding, and accelerate the breeding process of new variety. Therefore, core collection plays an important role in genetic diversity analysis and utilization, germplasm resource conservation, and crop genetic improvement, and is utilized in many horticultural crops [19,20,21], such as soybean [22], ginger [23], pea [24], peanut [25], tomato [26], and banana [27]. Early data used for constructing core collections of horticultural crops were phenotypic data, and core collection was constructed by detailed investigation of agronomic traits, although the data were susceptible to environmental factors and investigators. Molecular markers, especially Simple Sequence Repeats (SSRs), which have distinct advantages such as high polymorphism, broad genomic coverage, and stable results, are widely used [28,29,30,31], and may become an important technology for the construction of crop core collection.

Current research on garlic germplasm accessions mainly focuses on the comprehensive evaluation of agronomic traits, screening of elite resources, genetic diversity analysis using molecular markers, and population genetic structure analysis [32,33,34,35,36,37,38]. Although there have been reports on the construction of garlic core collections, a universally accepted method has not been established [39,40,41,42]. Therefore, to screen germplasm resources with rich variation types, high genetic diversity, and strong heterogeneity, this study investigated and analyzed 21 agronomic traits of 366 garlic germplasm resources. Three sampling methods (random, priority, and deviation) were used to construct core collections, and their representativeness was evaluated. The results provide a scientific basis for the establishment of a germplasm nursery, efficient conservation and utilization of germplasm resources, and the breeding of elite varieties of garlic.

## 2. Results

### 2.1. Genetic Diversity Analysis of the Agronomic Traits of Garlic

Among the 21 agronomic traits of garlic, 7 are qualitative, and their genetic diversity is shown in Table 1. The 7 qualitative traits have 23 variation types, with different distribution frequencies among traits. The range of the genetic diversity index (*H*′) was 0.48–1.11. Leaf color had the highest *H*′ (1.11), with rich variation types, mainly green, accounting for 58.20%. Clove uniformity had the lowest *H*′ (0.48), with uniform accounting for 84.97% of variation types. Plant type had an *H*′ of 0.71, with semi-erect accounting for 76.23%. Leaf straightness had an *H*′ of 0.55, with drooping accounting for 84.15%. Bulb shape had an *H*′ of 0.90, with oblate spheroid accounting for 56.28%. Clove arrangement had an *H*′ of 0.77, with four variations: regular two-whorled accounting for 65.03%, regular single-whorled accounting for 31.69%, and no single-headed type. Bulb disc position had an *H*′ of 0.95, with flat accounting for 59.56% and concave 22.13%.

The *H*′ values of the 14 quantitative traits of the 366 garlic germplasm accessions are listed in Table 2. The coefficient of variation (CV) values of the quantitative traits ranged from 12.30 to 37.49%. Leaf number per plant had the smallest CV (12.30%), while weight per bulb had the largest (37.49%). *H*′ ranged from 3.08 to 5.80. Leaf number per plant had the smallest *H*′ (3.08), and bulb transverse diameter had the largest (5.80). Ten quantitative traits had *H*′ values exceeding 5.00, indicating that these germplasm accessions have rich genetic diversity compared to previous studies [32,35].

### 2.2. Correlation Analysis of the Agronomic Traits of Garlic

The correlation analysis of the 14 quantitative traits of garlic showed that all were significantly or extremely significantly correlated (Table 3). Except for the significant correlation between clove number per bulb and plant height, and between clove number per bulb and clove back width, all other indices showed extremely significant correlations. Aerial pseudostem height, aerial pseudostem diameter, bulb height, bulb transverse diameter, and weight per bulb were the five traits with high breeding attention.

Among them, the correlation coefficient between aerial pseudostem height and plant height was the highest (0.726), followed by that between aerial pseudostem height and aerial pseudostem diameter (0.673). The top three indices with the highest correlation coefficients with aerial pseudostem diameter were leaf width (0.804), leaf length (0.769), and clove height (0.754). The top three indices with the highest correlation coefficients with bulb height were weight per bulb (0.889), clove height (0.875), and bulb transverse diameter (0.808). The top three indices with the highest correlation coefficients with bulb transverse diameter were weight per bulb (0.953), clove height (0.826), and bulb height (0.808). The top three indices with the highest correlation coefficients with weight per bulb were bulb transverse diameter (0.953), bulb height (0.889), and clove height (0.873).

The correlation coefficients between the aboveground parts (leaf length and leaf width) and belowground parts (bulb transverse diameter, weight per bulb, and bulb disc diameter) were all above 0.700. The correlation coefficients between aerial pseudostem diameter and the three underground traits (bulb height, weight per bulb, and clove height) were also above 0.700, indicating that leaf size and aerial pseudostem diameter significantly affect underground agronomic traits.

### 2.3. Principal Component Analysis of the Agronomic Traits of Garlic

Principal component analysis was performed on 21 agronomic traits of all accessions (Table 4). The results showed that the cumulative contribution rate of the first four principal components was 70.19%, indicating that these four principal components can represent most of the genetic information of the 21 traits. Among them, the first principal component was chiefly related to leaf, aerial pseudostem, and bulb traits and had an eigenvalue of 9.340, a contribution rate of 44.475%, and mainly included eight indices, namely, leaf length, leaf width, aerial pseudostem diameter, bulb height, bulb transverse diameter, weight per bulb, clove height, and bulb disc diameter, with eigenvector values all above 0.800. The second principal component was chiefly related to bulb shape and plant type traits and had an eigenvalue of 2.988, a contribution rate of 14.230%, and included four indices, namely, bulb shape, clove arrangement, plant type, and leaf straightness. The third principal component had an eigenvalue of 1.292, a contribution rate of 6.150%, and included three indices, namely, clove number per bulb, clove uniformity, and bulb disc position. The fourth principal component had an eigenvalue of 1.121, a contribution rate of 5.337%, and corresponded to leaf color.

### 2.4. Cluster Analysis of the Agronomic Traits of Garlic

**Figure 1:**
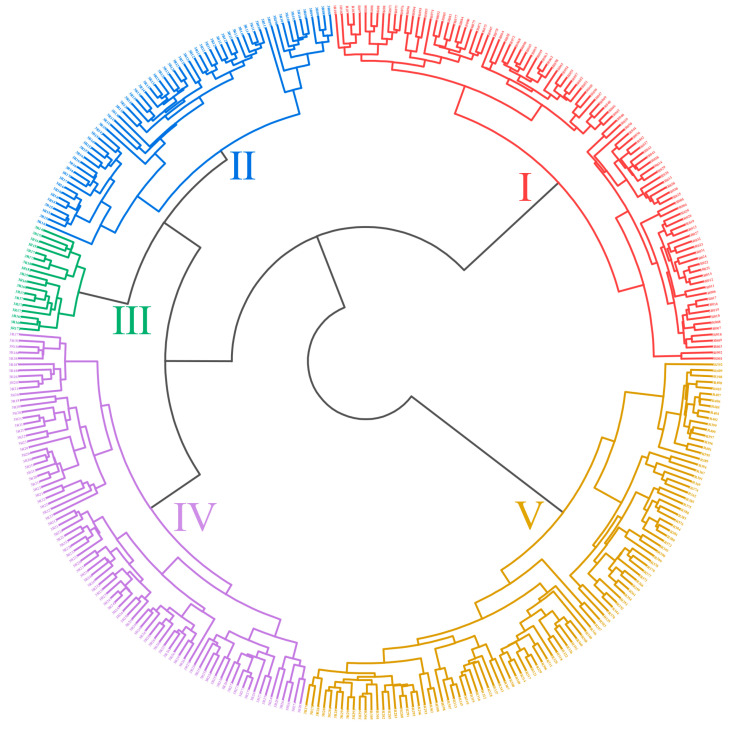
Cluster analysis of 366 garlic germplasm accessions based on 21 agronomic traits. All garlic accessions are numbered starting with “3R”, and the detail information was listed in Table S1.

The 366 garlic germplasm accessions were divided into five categories by cluster analysis based on 21 agronomic traits (Figure 1). Category I contained 97 accessions, accounting for 26.50% of the total resources, and this category had high values in indices such as plant height, plant breadth, aerial pseudostem height, aerial pseudostem diameter, bulb height, weight per bulb, clove height, and clove back width, indicating vigorous aboveground growth and presumably high bulb yield. The representative resource was 3R052. Category II contained 63 accessions, accounting for 17.21% of the total, and these resources had moderate values in indices such as leaf number per plant, plant height, aerial pseudostem height, bulb height, and bulb transverse diameter, but had wider leaves than those represented by category I resources. The representative resource was 3R135. Category III contained 18 accessions, accounting for 4.92% of the total. This category was characterized by compact and short plants, with generally low bulb indices, indicating small garlic bulbs. The bulb shape was mainly high spheroid, with irregular clove arrangements and straight leaves. The representative resource was 3R166. Category IV contained 86 accessions, accounting for 23.50% of the total. These resources had more leaves per plant, with larger leaves (leaf length and width indices), moderate plant height and breadth, and higher aerial pseudostem diameter, bulb transverse diameter, and bulb disc diameter indices. The plant type was mainly semi-erect, the bulb shape was mainly oblate spheroid, and the leaf color was mainly yellow-green. The representative resource was 3R228. Category V contained 102 accessions, accounting for the highest proportion (27.87% of the total). These resources had moderate plant size and bulb indices, clove arrangements that were mainly regular single-whorled, and bulb disc positions that were mainly concave. The representative resource was 3R343.

### 2.5. Construction and Evaluation of Garlic Core Collections

Genetic distance was calculated according to Euclidean distance using QGA Station 2.0 software, and clustering was performed by the unweighted pair group method with arithmetic mean (UPGMA). Three methods, namely, random (R), priority (P), and deviation (D), were applied to construct core collections for sampling at a 10% ratio, and their representativeness was evaluated. R1, P1, and D1 contained 37, 36, and 33 accessions, respectively. Among them, the number of repeated accessions was six between R1 and P1, four between R1 and D1, and seven between P1 and D1. Therefore, we constructed 90 garlic core collections in this study.

The percentage difference between core collections and the original population are showed in Table 5. Compared with the original population, the mean difference percentages (MD) of R1, P1, and D1 were 0.37%, 1.48%, and 13.04%, respectively, all below 20%. The range coincidence rates (CR) of R1 and P1 were 79.82% and 100%, respectively, indicating that R1 and P1 met the principles of core collection construction, can represent the genetic diversity of the original population, and reflect the genetic diversity of the original germplasm accessions. The mean t-test was used to examine differences in agronomic trait means between the original population and core collections, and the variance *F*-test was used to analyze the homogeneity of agronomic trait variation between the two groups [19]. Comparisons of mean and variance differences between core collections and original population are listed in Table 6. Core collection R1 had 13 traits with higher variance and 13 traits with higher coefficients of variation than those of the original population. Core collection P1 had 15 traits with significant or extremely significant variance differences from those in the original population and 20 traits with higher coefficients of variation. Core collection D1 had 16 traits with significant or extremely significant mean differences from those of the original population, indicating that the three core collections have heterogeneity, and P1 has the largest genetic variation.

## 3. Discussion

### 3.1. Genetic Diversity Analysis of Garlic Germplasm Accessions

As a vegetatively propagated crop, garlic cannot create variant offspring through sexual hybridization (neither naturally nor artificially). New variation types mainly rely on the accumulation of natural variations, microenvironmental adaptation, and artificial physical and chemical mutagenesis [38]. Similar to crops such as cassava and yam, which mainly spread through vegetative organs, the accumulation of natural variation is a long process, but still has important value. The accumulation of variation will generate new genotypes, and breeders will discover and utilize valuable genotypes to cultivate new varieties. During the spread of these vegetatively propagated crops, harmful variables are suppressed or eliminated through screening by breeders and growers, while beneficial variables are continuously selected and utilized. As a result, although these crops do not have strict geographical population structures, they have accumulated many beneficial variables, showing rich genetic diversity [43].

In this study, 366 selected garlic germplasm accessions from 10 countries showed rich genetic diversity. The coefficients of variation of aerial pseudostem height and aerial pseudostem diameter were both close to 20%, the highest among aboveground agronomic traits. Weight per bulb had the highest coefficient of variation among underground agronomic traits (37.49%), which was consistent with previous reports [34,35]. The correlation coefficient between aerial pseudostem diameter and weight per bulb (the important yield index) was 0.712, showing a very significant correlation. Therefore, in breeding new, high-yield garlic varieties, attention should be focused on the aerial pseudostem diameter index.

The *H*′ values of the seven qualitative traits were similar to those in previous reports, not exceeding 1.20. However, the *H*′ values of the 14 quantitative traits all exceeded 3.00, with 10 exceeding 5.00, which were significantly higher values than reported previously [32,35]. This indicated that the quantitative traits of garlic germplasm accessions used in this study addressed a very good representation of the genetic diversity present in the existing garlic germplasm accessions and the selected set of accessions seemed to have important values in environmental adaptability, evolutionary potential, and breeding utilization. Generally, geographical distribution is a crucial factor influencing the genetic diversity of garlic. Germplasm accessions with the same origin typically share similar genetic backgrounds and growth performances [44,45,46]. The clustering results of agronomic traits in this study also indicated that foreign garlic accessions from the same region (or country) tend to be clustered into the same group. For example, 3R175 and 3R176, both from the United States, were clustered in Category III; 3R267 and 3R227, both from India, were clustered in Category IV; 3R311 from Japan and 3R312 from South Korea were both clustered in Category V. However, 3R310 from the United States and 3R298 from Canada were also clustered in Category V. Other foreign garlic accessions were mainly clustered in Category IV (3R254 from Ukraine and 3R279 from Canada) and Category V (3R302 from Egypt and 3R307 from Australia). These non-China germplasm accessions have improved the overall genetic diversity to a certain degree, and this may be one reason for higher genetic diversity index (*H*′) of the agronomic traits observed in this study.

### 3.2. Construction and Evaluation of Garlic Core Collections

Previous studies on constructing garlic core collections have mainly used two types of data: agronomic traits and molecular markers. Due to the differences in the origins of garlic accessions, the number of selected accessions, the number of agronomic traits, and the types of molecular markers, the results of different studies have varied greatly. The Leibniz Institute of Plant Genetics and Crop Plant Research (IPK) in Germany conserved 160 garlic collections, representing 25% of the total accessions, and the data contained 15 phenotypic traits and a comprehensive set of images for each accession [39]. A core set of 46 accessions was selected from 625 Indian garlic accessions based on thirteen quantitative and five qualitative traits, representing 7.36% of the total accessions [40]. Eight SSR (Simple Sequence Repeats) markers were used to construct core collection, a set of 95 accessions was successfully developed from 613 garlic accessions, and the proportion of the core collection is 15.50% [41]. Through the GBS (genotyping-by-sequencing) method, 286 core collections were constructed from 417 garlic germplasm accessions conserved in Spain, and the core collection proportion is 68.59% [42]. Therefore, while a universally accepted method for constructing garlic core collection has not been established, these studies provided important value for understanding the genetic diversity of world garlic germplasm accession.

To evaluate core collections, four indices are usually used: mean difference percentage, variance difference percentage, range coincidence rate, and coefficient of variation change rate [47,48]. The smaller the mean difference percentage and the larger the variance difference percentage, range coincidence rate, and coefficient of variation change rate, the better the representativeness of the constructed core collection [49]. Although the mean difference percentage of P1 was not the lowest, its other three indices were the highest among the three groups, indicating that P1 had the best representativeness and largest genetic variation among the three core collections constructed from agronomic trait data in this study. The heterogeneity of core collection construction results was enhanced by the different focuses of the three sampling methods. To retain as much genetic diversity of the original accessions as few accessions as possible, 90 germplasm accessions, accounting for 24.59% of the total 366 collections, were screened out of the garlic core collections in this study by removing redundant resources.

## 4. Materials and Methods

### 4.1. Experimental Materials

**Figure 2:**
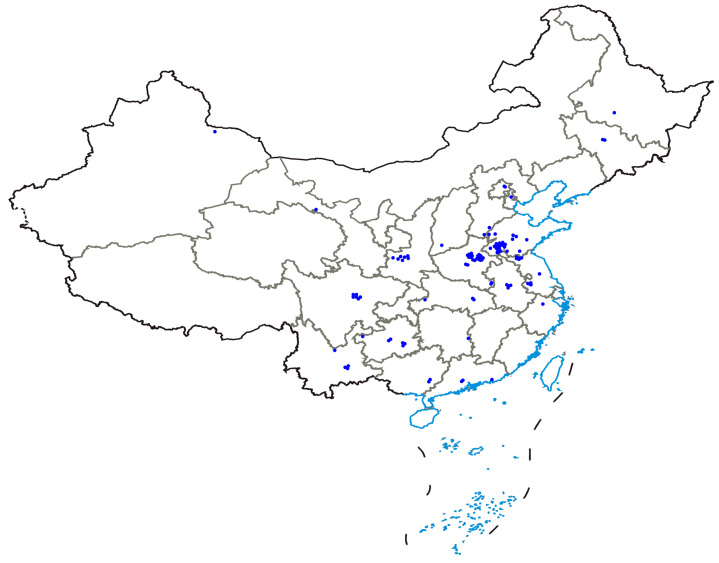
The distribution of Chinese garlic germplasm accessions used in this article.

The 366 garlic germplasm accessions used in this study were conserved by our team. These resources were sourced from 10 countries, including 352 accessions from 21 provinces in China (accounting for 96.17% of the total accessions), and 14 accessions from 9 other countries (3.83% of the total). Garlic accessions from China were mainly collected from major garlic-producing areas, such as Shandong, Henan, Jiangsu, Shaanxi, Sichuan, Guizhou, Anhui, and Yunnan, and the distributions of Chinese garlic germplasm accessions are listed in Figure 2. All resources were numbered starting with “3R”, and their detailed information is listed in Table S1.

Planting was carried out in early October 2023 at the Henan Modern Agricultural Research and Development Base (35°0′58″ N, 113°42′42″ E, H: 67 m). Plants were spaced 15 cm apart, and rows were spaced 20 cm apart. The plot area was approximately 4.5 m^2^, with 3 replicates. The planting soil was sandy loam, and field management measures, including water and fertilizer management and pest control, were carried out according to conventional methods [35].

### 4.2. Investigation of Agronomic Traits

From April to June 2024, referring to *Descriptors and Data Standards for Garlic Germplasm Resources* [50], 21 agronomic traits were investigated, including 14 quantitative traits: leaf number per plant, leaf length, leaf width, plant height, plant breadth, aerial pseudostem height, aerial pseudostem diameter, bulb height, bulb transverse diameter, weight per bulb, clove height, clove back width, clove number per bulb, bulb disc diameter; and 7 qualitative traits: plant type, leaf straightness, leaf color, bulb shape, clove arrangement, clove uniformity, and bulb disc position [45]. For quantitative traits, 10 plants were set as one biological replicate, and for qualitative traits, 6 plants were set as one biological replicate; three biological replicates were set for each germplasm accession. These indices were measured using a meter ruler, Vernier caliper, and electronic balance.

### 4.3. Data Analysis

We used Excel 2021 (Microsoft, Redmond, WA, USA) software to summarize the agronomic traits of the garlic germplasm accessions and calculate the means and coefficients of variation of quantitative traits. Principal component and correlation analysis were conducted using SPSS 17.0 software (IBM, Armonk, NY, USA). According to the method reported by Wang [51], each agronomic trait was graded quantitatively, and the Shannon diversity index (*H*′) of each trait was calculated by the formula *H*′ = −∑Pi lnP_i_, where i is the grade of a certain trait and P_i_ is the frequency of samples in the ith grade, i.e., the frequency in the population.

### 4.4. Construction of Core Collections

Genetic distance was calculated according to Euclidean distance using QGA Station 2.0 software (Zhejiang University Institute of Bioinformatics, Hangzhou, China) and clustering was performed by UPGMA. To conserve the genetic diversity to the greatest extent possible, three sampling methods were used to construct the garlic core collection: (1) random (R), which randomly selects accessions at a 5–20% proportion from the total collections, and suits preliminary screening, but may miss rare variations; (2) priority (P), which prioritizes and selects accessions with key parameters (e.g., agronomic traits or rare alleles) and can retain critical diversity for specific purposes; and (3) deviation (D), which calculates the “deviation degree” of each accession and then preferentially selects accessions with larger deviations and can capture rare or distinctive accessions [20].

### 4.5. Evaluation of Core Collection Representativeness

Referring to the method reported by Hu et al. [49], the construction of core collections must meet two conditions simultaneously: (1) the percentage of traits with significant differences (α = 0.05) in mean from the original germplasm resource population must be less than or equal to 20%; (2) the range coincidence rate between the core collection and the original germplasm resource population must not be less than 80%. The range coincidence rate (CR), variation rate of the coefficient of variation (VR), mean difference percentage (MD), and variance difference percentage (VD) were used to evaluate the representativeness of the core collections. The mean *t*-test of genetic parameters was used to test the differences in phenotypic trait means between the original population and the core collections, and the variance *F*-test was used to analyze the homogeneity of phenotypic trait variations between the two groups [52].

## 5. Conclusions

In this study, 21 agronomic traits of 366 garlic germplasm accessions were investigated and statistically analyzed, including by genetic diversity, correlation, principal component, and cluster analyses, revealing that these resources have rich genetic diversity. Three core collections (R1, P1, and D1) were constructed using random (R), priority (P), and deviation (D) sampling. Among the constructed core collections, R1 and P1 met the necessary requirements and reflect the genetic diversity of the original population. D1 had 16 traits with significant or very significant mean differences from those of the original population, indicating that the three core collections have good heterogeneity, and P1 had the largest genetic variation. To retain as much genetic diversity of the original population as possible, 90 garlic core collections were constructed after removing redundant resources, ultimately accounting for 24.59% of the original population. These core collections lay an important foundation for the conservation of garlic genetic diversity, the exchange and utilization of germplasm accessions between countries, and the establishment of a germplasm nursery for garlic.

## Figures and Tables

**Table 1:** Genetic diversity analysis of qualitative traits of 366 garlic germplasm accessions.

Trait	Genetic Diversity Index (*H*′)	Distribution Frequency/%				
		1	2	3	4	5
PT	0.71	14.48	76.23	9.29	—	—
LS	0.55	84.15	6.83	9.02	—	—
LC	1.11	11.75	21.31	58.20	8.74	—
BP	0.90	56.28	34.97	8.74	—	—
CA	0.77	0.55	65.03	31.69	—	2.73
CU	0.48	84.97	13.11	1.91	—	—
BDP	0.95	18.31	59.56	22.13	—	—

PT: Plant type. LS: Leaf straightness. LC: Leaf color. BP: Bulb shape. CA: Clove arrangement. CU: Clove uniformity. BDP: Bulb disc position. Plant type: 1. Erect; 2. Semi-erect; 3. Spreading. Leaf straightness: 1. Drooping; 2. Semi-drooping; 3. Straight. Leaf color: 1. Yellowish green; 2. Light green; 3. Green; 4. Dark green. Bulb shape: 1. Oblate spheroid; 2. Sub-spheroid; 3. High spheroid. Clove arrangement: 1. Regular multi-whorled; 2. Regular two-whorled; 3. Regular single-whorled; 4. Single-headed; 5. Irregular. Clove uniformity: 1. Uniform; 2. Relatively uniform; 3. Non-uniform. Bulb disc position: 1. Convex; 2. Flat; 3. Concave. “—”: This variation type not detected. The numbers 1 to 5 in the “Distribution frequency/%” column represent the variation types of each trait.

**Table 2:** Genetic diversity analysis of quantitative traits of 366 garlic germplasm accessions.

Trait	Mean	Maximum	Minimum	Standard Deviation	Coefficient of Variation/%	Genetic Diversity Index (*H*′)
LN	6.84	8.83	3.50	0.84	12.30	3.08
LL/cm	51.68	67.58	22.85	7.30	14.13	5.71
LW/cm	2.85	4.40	1.25	0.43	15.06	4.42
PH/cm	52.27	77.50	27.60	9.19	17.58	5.03
PB/cm	53.91	77.68	20.00	10.20	18.93	5.16
APH/cm	24.49	37.50	8.50	4.79	19.58	4.57
APD/cm	14.08	22.85	5.05	2.68	19.05	5.69
BH/mm	30.11	40.17	15.42	4.55	15.11	5.78
BTD/mm	49.14	63.86	22.12	8.08	16.43	5.80
WPB/g	44.70	82.40	6.50	16.76	37.49	5.66
CH/mm	27.94	36.15	14.77	4.03	14.44	5.74
CBW/mm	15.37	24.34	8.56	2.44	15.88	5.65
CNPB	10.14	18.33	3.17	1.64	16.14	3.41
BDD/mm	19.56	25.89	8.51	2.91	14.89	5.64

LN: Leaf number per plant. LL: Leaf length. LW: Leaf width. PH: Plant height. PB: Plant breadth. APH: Aerial pseudostem height. APD: Aerial pseudostem diameter. BH: Bulb height. BTD: Bulb transverse diameter. WPB: Weight per bulb. CH: Clove height. CBW: Clove back width. CNPB: Clove number per bulb. BDD: Bulb disc diameter.

**Table 3:** Correlation analysis of quantitative traits of 366 garlic germplasm accessions.

Trait	LN	LL	LW	PH	PB	APH	APD	BH	BTD	WPB	CH	CBW	CNPB	BDD
LN	1													
LL	0.470 **	1												
LW	0.485 **	0.764 **	1											
PH	0.485 **	0.475 **	0.378 **	1										
PB	0.206 **	0.695 **	0.644 **	0.179 **	1									
APH	0.464 **	0.576 **	0.538 **	0.726 **	0.229 **	1								
APD	0.504 **	0.769 **	0.804 **	0.569 **	0.603 **	0.673 **	1							
BH	0.415 **	0.643 **	0.661 **	0.421 **	0.502 **	0.596 **	0.704 **	1						
BTD	0.398 **	0.750 **	0.769 **	0.308 **	0.618 **	0.564 **	0.694 **	0.808 **	1					
WPB	0.424 **	0.703 **	0.728 **	0.354 **	0.559 **	0.608 **	0.712 **	0.889 **	0.953 **	1				
CH	0.375 **	0.690 **	0.692 **	0.410 **	0.550 **	0.597 **	0.754 **	0.875 **	0.826 **	0.873 **	1			
CBW	0.378 **	0.493 **	0.530 **	0.404 **	0.315 **	0.513 **	0.563 **	0.621 **	0.622 **	0.671 **	0.658 **	1		
CNPB	0.213 **	0.345 **	0.368 **	0.129 *	0.266 **	0.320 **	0.262 **	0.461 **	0.508 **	0.474 **	0.316 **	0.105 *	1	
BDD	0.346 **	0.726 **	0.708 **	0.205 **	0.642 **	0.398 **	0.620 **	0.690 **	0.878 **	0.810 **	0.694 **	0.497 **	0.475 **	1

LN: Leaf number per plant. LL: Leaf length. LW: Leaf width. PH: Plant height. PB: Plant breadth. APH: Aerial pseudostem height. APD: Aerial pseudostem diameter. BH: Bulb height. BTD: Bulb transverse diameter. WPB: Weight per bulb. CH: Clove height. CBW: Clove back width. CNPB: Clove number per bulb. BDD: Bulb disc diameter. Single asterisk (*) and double asterisks (**) mean significant and very significant correlations at the 0.05 and 0.01 probability levels, respectively.

**Table 4:** Principal component analysis of agronomic traits of 366 garlic germplasm accessions.

Trait	Principal Component			
	1	2	3	4
LN	0.493	0.463	−0.062	0.120
LL	0.836	0.111	−0.082	0.310
LW	0.852	0.056	−0.043	0.172
PH	0.435	0.726	−0.148	0.172
PB	0.693	−0.236	−0.097	0.337
APH	0.651	0.526	−0.029	−0.029
APD	0.830	0.276	−0.117	0.153
BH	0.869	0.128	0.085	−0.175
BTD	0.946	−0.093	0.100	−0.044
WPB	0.943	−0.007	0.090	−0.181
CH	0.894	0.055	−0.104	−0.185
CBW	0.667	0.262	−0.005	−0.334
CNPB	0.467	−0.017	0.588	0.197
BDD	0.853	−0.181	0.114	0.142
BP	−0.490	0.502	−0.035	−0.050
CA	−0.443	0.504	0.212	−0.136
CU	−0.139	0.395	0.636	−0.184
BDP	0.076	−0.341	0.614	0.252
PT	0.369	−0.677	−0.034	−0.101
LS	−0.548	0.676	0.081	0.224
LC	0.515	−0.005	0.057	−0.604
Eigen value	9.340	2.988	1.292	1.121
Contribution rate %	44.475	14.230	6.150	5.337
Cumulative contribution rate %	44.475	58.705	64.855	70.192

LN: Leaf number per plant. LL: Leaf length. LW: Leaf width. PH: Plant height. PB: Plant breadth. APH: Aerial pseudostem height. APD: Aerial pseudostem diameter. BH: Bulb height. BTD: Bulb transverse diameter. WPB: Weight per bulb. CH: Clove height. CBW: Clove back width. CNPB: Clove number per bulb. BDD: Bulb disc diameter. BP: Bulb shape. CA: Clove arrangement. CU: Clove uniformity. BDP: Bulb disc position. PT: Plant type. LS: Leaf straightness. LC: Leaf color.

**Table 5:** Percentage difference between core collection and initial accessions.

Percentage Difference %	Core Collection		
	R1	P1	D1
Coincidence rate of range/% CR	79.82	100	56.28
Variation rate of coefficient of variation/% VR	101.72	144.3	59.6
Mean difference percentage/% MD	0.37	1.48	13.04
Variance difference percentage/% VD	4.9	96.67	52.58

R1, P1, and D1 represent the core collections constructed by random sampling method, preferential sampling method, and deviation sampling method, respectively.

**Table 6:** Difference comparisons between core collection and initial accessions.

Trait	Parameter	Initial Population	Core Collection		
			R1	P1	D1
LN	Mean	6.84	6.87	6.77	7.80 **
	Variance	0.70	0.64	1.25 **	0.28
	CV/%	12.30	11.69	16.73	6.91
LL	Mean/cm	51.68	51.39	48.57	59.19 **
	Variance	53.16	60.44	136.05 **	16.36
	CV/%	14.13	15.13	24.35	6.94
LW	Mean/cm	2.85	2.80	2.68	3.37 **
	Variance	0.18	0.18	0.47	0.07
	CV/%	15.06	15.28	25.91 **	7.94
PH	Mean/cm	52.27	52.41	51.78	58.30 **
	Variance	84.24	90.33	156.58 **	24.08
	CV/%	17.58	18.13	24.51	8.55
PB	Mean/cm	53.91	54.12	49.24	59.53 *
	Variance	103.83	123.81	260.25 **	31.42
	CV/%	18.93	20.56	33.23	9.56
APH	Mean/cm	24.49	24.32	23.05	29.32 **
	Variance	22.92	25.59	47.16 **	10.04
	CV/%	19.58	20.80	30.21	10.97
APD	Mean/cm	14.08	13.83	13.58	17.44 **
	Variance	7.17	7.33	19.72 **	3.62
	CV/%	19.05	19.58	33.17	11.09
BH	Mean/mm	30.11	30.41	28.25	34.81 **
	Variance	20.63	26.95	41.04 **	8.07
	CV/%	15.11	17.07	23.00	8.29
BTD	Mean/mm	49.14	48.84	45.18	58.22 **
	Variance	65.03	65.72	143.76 **	6.64
	CV/%	16.43	16.60	26.91	4.49
WPB	Mean/g	44.70	45.02	39.57	65.93 **
	Variance	280.09	301.86	492.43 **	52.50
	CV/%	37.49	38.60	56.87	11.16
CH	Mean/mm	27.94	28.05	26.29	32.03 **
	Variance	16.22	16.21	33.84 **	4.40
	CV/%	14.44	14.35	22.44	6.65
CBW	Mean/mm	15.37	15.87	15.52	18.46 **
	Variance	5.95	7.11	11.17 **	3.13
	CV/%	15.88	16.81	21.83	9.73
CNPB	Mean	10.14	10.24	9.73	10.86 *
	Variance	2.67	2.91	8.04 *	1.28
	CV/%	16.14	16.67	29.56	10.56
BDD	Mean/mm	19.56	19.41	17.90	22.53 **
	Variance	8.46	7.75	19.12 **	2.18
	CV/%	14.89	14.34	24.77	6.65
PT	Mean	1.95	1.95	1.72	2.00
	Variance	0.24	0.22	0.26	0.06
	CV/%	24.92	24.06	29.81	12.50
LS	Mean	1.25	1.27	1.47	1.03 **
	Variance	0.37	0.37	0.58 *	0.03
	CV/%	48.59	47.84	52.58	16.90
LC	Mean	2.64	2.57	2.69	3.52 **
	Variance	0.64	0.59	0.82 **	0.37
	CV/%	30.36	29.80	34.15	17.60
BP	Mean	1.52	1.62	1.69	1.39
	Variance	0.42	0.52	0.49	0.36
	CV/%	42.78	44.45	41.90	43.71
CA	Mean	2.39	2.49	2.61	2.33
	Variance	0.41	0.42	0.79	0.40
	CV/%	26.90	26.17	34.59	27.66
CU	Mean	1.17	1.16	1.31	1.30
	Variance	0.18	0.14	0.32	0.27
	CV/%	36.22	32.15	44.17	40.63
BDP	Mean	2.04	2.00	2.11	2.12
	Variance	0.40	0.44	0.49	0.35
	CV/%	31.18	33.33	33.55	28.28

LN: Leaf number per plant. LL: Leaf length. LW: Leaf width. PH: Plant height. PB: Plant breadth. APH: Aerial pseudostem height. APD: Aerial pseudostem diameter. BH: Bulb height. BTD: Bulb transverse diameter. WPB: Weight per bulb. CH: Clove height. CBW: Clove back width. CNPB: Clove number per bulb. BDD: Bulb disc diameter. PT: Plant type. LS: Leaf straightness. LC: Leaf color. BP: Bulb shape. CA: Clove arrangement. CU: Clove uniformity. BDP: Bulb disc position. R1, P1 and D1 represent the core collections constructed by random sampling method, preferential sampling method and deviation sampling method, respectively. CV: Coefficient of variation. Single asterisk (*) and double asterisks (**) represent significant and highly significant differences for the mean or variance between the core collection and initial population with the same trait at the 0.05 and 0.01 levels.

## Data Availability

The data presented in this study are available on request from the corresponding author due to the funding agency approval.

## Supplementary Materials

The following supporting information can be downloaded at: https://www.mdpi.com/article/10.3390/plants14193009/s1, Table S1: Detailed information on the 366 garlic germplasm resources.

## Abbreviations

The following abbreviations are used in this manuscript:

CR	Range coincidence rate
CV	Coefficient of variation
*H*′	Genetic diversity index
MD	Mean difference percentage
SSR	Simple sequence repeat
UPGMA	Unweighted pair group method with arithmetic mean
